# Reconfigurable Mach–Zehnder interferometer for dynamic modulations of spoof surface plasmon polaritons

**DOI:** 10.1515/nanoph-2021-0539

**Published:** 2021-12-01

**Authors:** Wen Yi Cui, Jingjing Zhang, Xinxin Gao, Tie Jun Cui

**Affiliations:** State Key Laboratory of Millimeter Waves, Southeast University, Nanjing 210096, China; Institute of Electromagnetic Space, Southeast University, Nanjing 210096, China; Pazhou Laboratory, Center for Intelligent Metamaterials, Guangzhou 510330, China

**Keywords:** amplitude modulation, frequency modulation, Mach–Zehnder interferometer, reconfigurable device, spoof surface plasmon polaritons

## Abstract

We propose an ultrathin reconfigurable Mach–Zehnder interferometer (MZI) for realizing dynamic frequency and amplitude modulations of spoof surface plasmon (SSP) signal. Active varactor diodes are integrated in the SSP unit cells on one of the MZI arms to introduce asymmetry to the MZI structure, which can control the interference patterns by varying bias voltages applied on the varactor diodes. We show that the spectral positions of multiple sharp interference dips are very sensitive to the change of diode capacitance, thereby allowing for good frequency modulation. We also demonstrate continuous amplitude modulation by tuning the varactor diodes at multiple selected frequencies. To verify the reconfigurable feature of the proposed SSP MZI, the frequency shift keying (FSK) and amplitude modulations have been experimentally demonstrated on the same structure. The modulation depth of the amplitude modulation can be further improved by designing geometrical parameters of the SSP structure, reaching a significant amplitude change from 0.88 to 0.05 in experiments.

## 1 Introduction

Surface plasmon polaritons (SPPs) are highly localized surface waves that propagate along the interface between two materials whose real parts of permittivity are positive and negative (such as air and metal in the optical frequency), respectively. The electromagnetic energy of the SPP wave propagating along the interface is confined in a deep subwavelength range, and decays exponentially in the direction perpendicular to the interface [1]. This distinctive characteristic contributes to the integration and miniaturization of the optical components and circuits. However, at microwave frequencies, the metal no longer behaves like a plasma with the negative permittivity but should be taken as a perfectly electric conductor. The electromagnetic fields cannot penetrate into the metal, and the metal–air interface can no longer support SPP waves with strong confinements of surrounding electromagnetic fields.

The birth of spoof surface plasmon polaritons (SSPPs) solved the problem. Pendry et al. found that punching subwavelength holes on the surface of a perfect conductor allows the field penetration, mimicking the SPPs in real surface plasmons. The designed subwavelength structure can be seen as an equivalent medium to imitate the plasma medium in nature, where the size and spacing of the holes determine the effective plasma frequency in Drude model, leading to the dispersion of SSPPs [2]. After that, Shen et al. proposed a nearly zero-thickness comb-shaped metallic strip which is flexible and conformal [3] as the alternative to the three-dimensional (3D) bulk structure to support the spoof surface plasmons, paving the way for applications of SSPP devices in integrated circuits. A variety of passive SSPP devices based on the ultrathin platform have been proposed afterward, such as filters, couplers, splitters and antennas [4], [5], [6], [7], [8], [9], [10], [11], [12], [13], [14], [15], [16], [17]. The unique ability of SSPP in subwavelength confinement of electromagnetic waves allows for significantly improving the performance of wireless communication system, providing solutions for device miniaturization, signal integrity, and reduction of mutual interference between channels [18], [19], [20]. However, the modern communication systems often work at multi-frequency bands or under multiple standards, giving rise to high demands in multi-frequency, multi-mode and tunable/reconfigurable devices.

The physical characteristics of passive SSPP devices are determined by their geometry structures, and thus difficult to be modulated dynamically. The proposal of active SSPP device can overcome this inflexibility by incorporating active elements, such as diode, transistor, and state-change medium, into the SSPP structures. Combining the SSPP structures with different active components such as external active circuits (e.g., SRR loaded with diode) [21], active chips [22], [23] and two-dimensional (2D) materials (e.g., amplifier) [24], different controllable functions can be realized, including the real-time control of frequency spectra [21], broadband amplification with high gain at around 20 dB [22], second-harmonic generation (SHG) with high efficiency [23] and dynamic manipulation of attenuation, transmission, and amplification [24].

The SSPP structure itself can also provide more space and freedom to integrate with the active elements than the traditional transmission line. In 2017, Zhang et al. firstly introduced varactor diode into the SSPP unit and evaluated the topological model of this process. The whole SSPP system can realize three digital-analog functionalities including logic gate, digital phase shifter and controllable slow-wave generations [25]. In the same year, Zhang et al. analyzed the loss mechanism of resistor loaded on the SSPP unit, providing a new method to predict the attenuation of lossy active SSPP devices [26]. Based on the active SSPP units, researchers have proposed various devices to achieve versatile functions [27], [28], [29], [30], [31], [32], such as band-pass tunable filter [27], and SHGs in both forward and backward modes [28]. Moreover, the tunability of active SSPP structures enables the integration of multiple functions on a single device. Gao et al. proposed a multi-functional device composed of two active SSPP waveguides and a substrate integrated waveguide (SIW) [29], where the control of the cutoff frequencies enables the device to switch between a power divider and a band-pass filter. The designed device in [30] can be switched in real time to realize transmission, unequal coupling, 3 dB direction coupling, and crossover transmission. More recently, Zhang et al. have used PIN diode as the state-change medium to control the effective tooth height of the SSPP unit and then change the dispersion state, providing possibilities for 2 amplitude shift keying (2ASK) and 2 phase shift keying (2PSK) modulations [31]. However, the SSPP waveguide is a low-pass filter, and 2 frequency shift keying (2FSK) modulation cannot be realized by only adjusting its cutoff frequency. Later, Zhang et al. introduced through holes to the ground in the outermost SSPP teeth, which makes the whole waveguide in one state become band-pass, and further achieves the 2FSK modulation at single frequency band [32].

In this work, we propose an active and reconfigurable Mach–Zehnder interferometer (MZI) to enhance the adjustability of the SSPP signals. MZI can transfer tiny phase difference between the SSPP waves propagating on the two arms into prominent transmission amplitude interference [33]. By manipulating the bias voltages applied on the varactor diodes embedded on the SSPP units in one arm, the capacitances of the diodes are modified, leading to the phase difference of the SSPP waves from two arms, and ultimately pronouncing interference dips at the output end of MZI. As the spectral positions of the interference dips are very sensitive to the diode capacitance, the SSPP MZI can be leveraged to achieve the 2FSK modulations. The multiple dips with high quality factors provide extended freedom in the frequency selection and hold promise for enhancing the frequency-band utilization ratio. More importantly, the reconfigurable SSPP MZI structure can also achieve a wide range of continuous amplitude modulations at multiple frequencies. In contrast to the 2ASK functions in [31] and [32] which only have two states of “on” and “off” at a certain frequency, our design offers the possibility to realize multi-bit communications at different frequency channels. The experiments prove that our SSPP MZI structure enables high modulation depth, where the transmission coefficient can change from 0.05 to 0.88 at the selected frequency.

## 2 Manipulation of dispersion property of the SSPP structure

**Figure 1:**
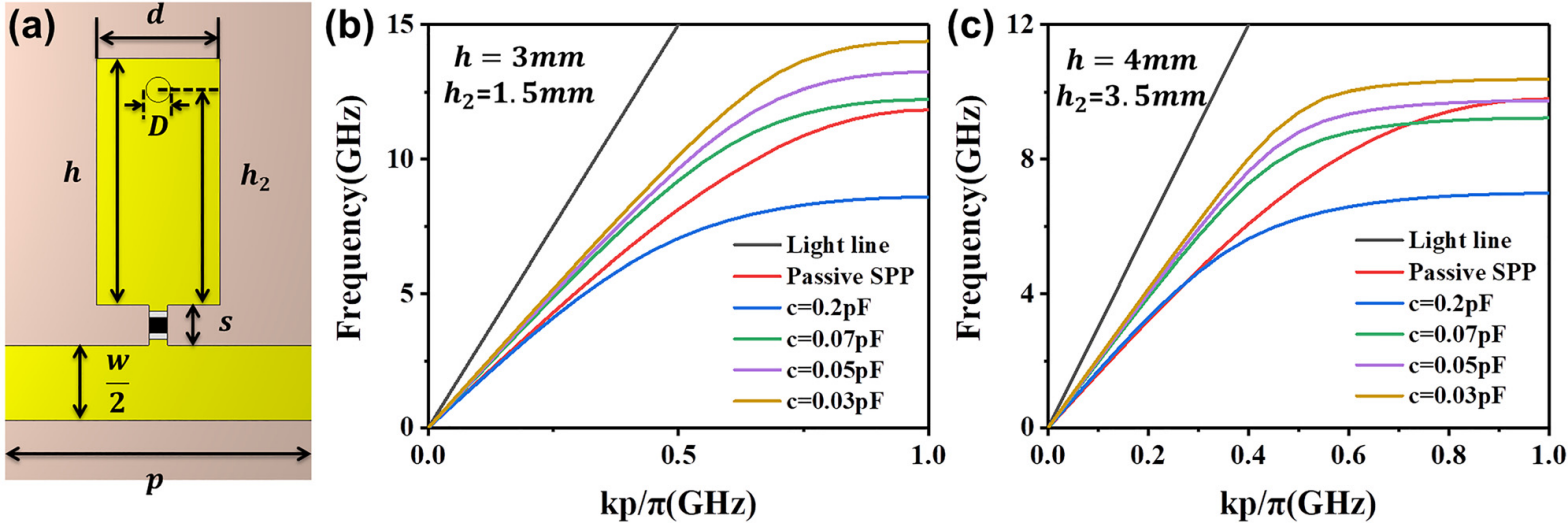
The active SSPP structure and its dispersion properties. (a) The active SSPP unit cell. (b)–(c) The simulated dispersion curves of the passive SSPP unit and active SSPP unit with (b) *h* = 3 mm and *h*_*2*_ = 1.5 mm, and (c) *h* = 4 mm and *h*_*2*_ = 3.5 mm, in which the capacitance *c* = 0.03, 0.05, 0.07 and 0.2 pF, respectively.

**Figure 2:**
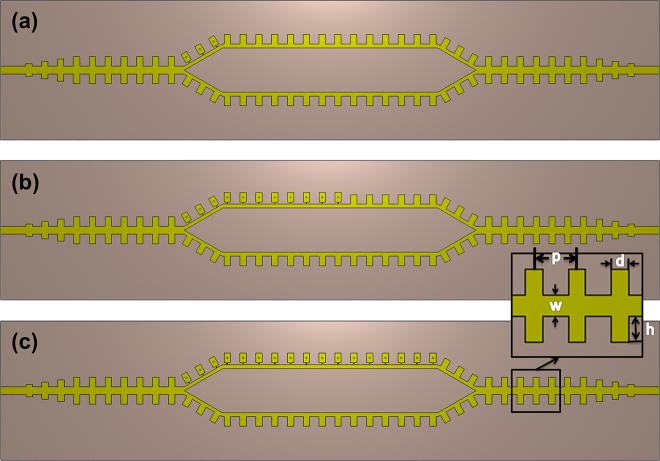
The active and reconfigurable SSPP-based MZI structures with (a) 3, (b) 11, and (c) 17 diodes in the upper arm.

We propose a reconfigurable SSPP MZI, in which varactor diodes are embedded in the SSPP unit cell on the upper arm to produce phase difference between the SSPP waves on the two arms, as shown in Figures 1(a) and 2. A through hole is punched in the SSPP tooth to connect it to the ground, so that the bias voltage can be applied on the diode. Here, we adopt the commercial diode MAVR-011020-1411, whose ideal capacitance ranges from 0.025 to 0.275 pF. We investigate the impact of varactor diodes on the dispersion performance of the SSPP structure, in which we set the capacitance of the diode model from 0.03 to 0.2 pF in the simulations. In addition, the inductance *L* is set as 0.5 nH and the resistance *R* is set as 2.5 Ω. We use Rogers RT 5880 as the substrate, whose thickness *t* = 0.787 mm, relative permittivity *ε*_r_ = 2.2, and loss tangent is 0.0009. To achieve the impedance matching at MZI ports, the line width *w* at the ports is set as 2.45 mm and the line width of the MZI arm is half of *w*. The spacing *s* between the tooth and strip is 0.65 mm to fit the size of the varactor diode, and the diameter *D* of the through hole is 0.4 mm. We remark that the passive SSPP unit does not have the spacing s and the through hole.

We use commercial software, CST Microwave Studio, to obtain the dispersion curves of the passive and active SSPP units with different geometrical parameters, as shown in Figure 1(b) and (c). We note that the load of the varactor diode and through hole can significantly change the dispersion property, and different propagation constants in the passive and active units will bring phase difference between the SSPP waves on the MZI arms with and without the active elements. The two figures also show that increasing the diode capacitance will lead to the decrease of the cut-off frequency. In other words, the dispersion curve will be farther away from the light line, indicating stronger confinement ability but shorter propagation distance. The change in the dispersion property also leads to the change in phase difference and finally alters the whole transmission. The capacitance can be modulated dynamically by adjusting the applied voltage so that the transmission properties can be engineered as desired. During parameter sweeping, we notice that the geometrical parameters not only affect the dispersion relations directly, but also affect the way that the diode capacitance influence the dispersion property, as depicted in Figure 1(b) and (c). Therefore, the geometrical parameters need to be judiciously chosen to ensure that the active and reconfigurable MZI device shows the optimized performance with large tuning range, according to different requirements in the practical applications.

## 3 Impact of active unit number on the transmission property of SSPP MZI

The above simulation is based on an SSPP unit cell with the periodic boundary condition to mimic the case of infinite number of unit cells. However, in the real applications, the varactor diodes will inevitably induce losses which will deteriorate the performance of the device. Therefore, we need to consider the trade-off between tunability and loss, and find out the maximum number of the varactor diodes which gives rise to the optimal modulation performance.

We consider three cases as shown in Figure 2, where the upper arm of the SSPP MZI has 3, 11, and 17 active SSPP units, respectively, and the lower arm consists of only passive SSPP units, and compare their transmission characteristics. The geometrical parameters of the SSPP MZI are the same as those used in Figure 1(b). We find that in a certain range, increasing the active units can significantly deepen the interference dips to optimize the modulation. However, further increasing the number of active units will offer little improvement in the tunability, and the loss induced by the diodes will outweigh the positive impacts brought by the extra active elements.

**Figure 3:**
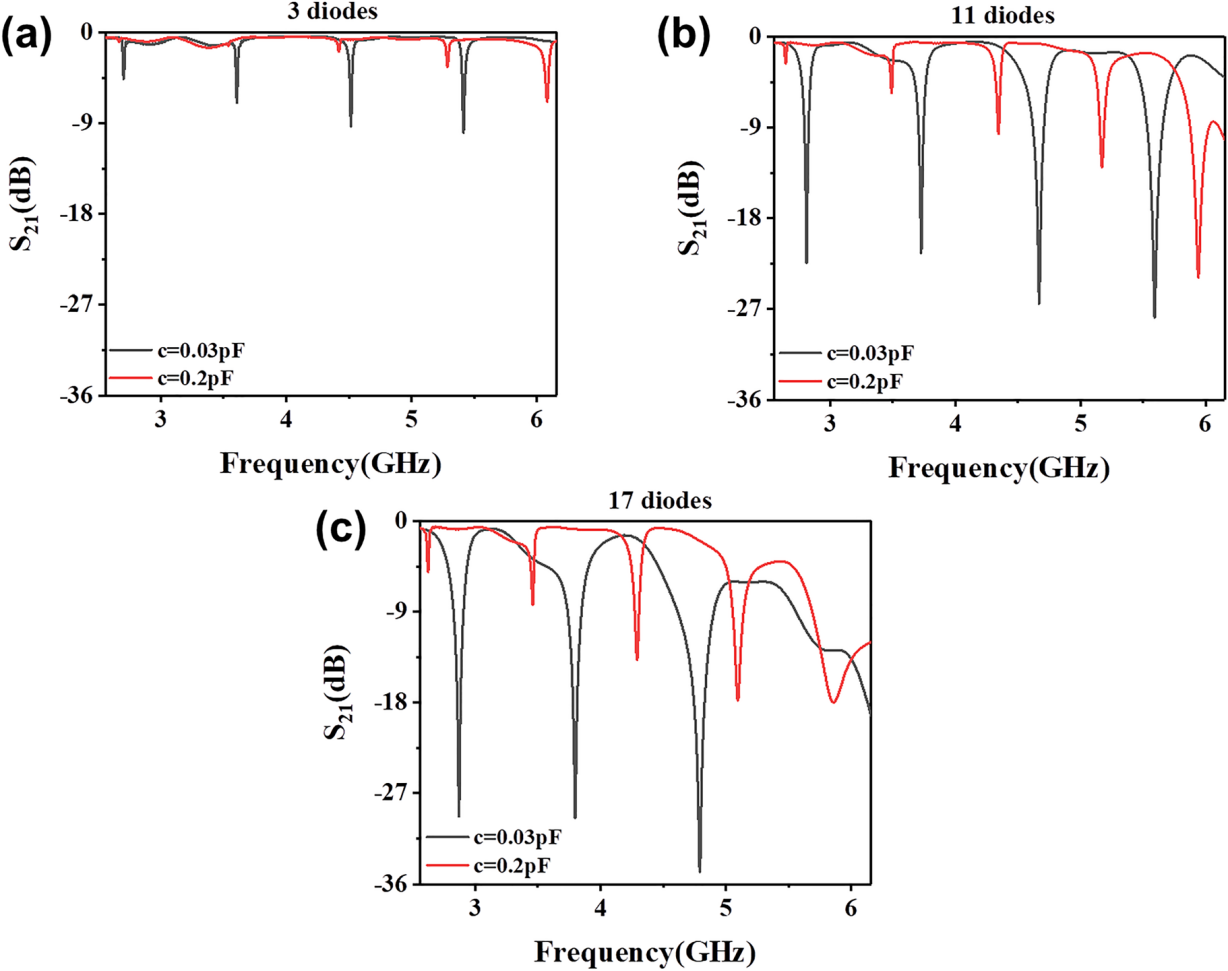
S_21_ curves of the SSPP MZI structure with (a) 3, (b) 11 and (c) 17 varactor diodes embedded on the upper arm.

Figure 3 illustrates the transmission coefficient curves of the three typical cases, where the SSPP MZI structures have 3, 11 and 17 diodes, respectively. We observe that when the number of the diode is only 3, all the transmission dips are above −10 dB, indicating that the EM signals can be transmitted in the whole frequency ranges from 2.5 to 6.5 GHz. Increasing the number of diodes will lead to deeper interference dips but smaller cutoff frequency as well. Moreover, the frequency shift of the interference dips with variant diode capacitance also increases with the diode number. When the number is as high as 17, the loss of the diodes will significantly deteriorate the transmission performance in the passband, particularly at higher frequencies. Considering the tradeoff among all factors that influence the frequency modulation, i.e., the transmission properties of the passbands and dips, and the frequency shift values, we find that 11 diodes give the optimal performance as desired, where 2FSK can be realized at multiple frequencies.

## 4 Simulation analysis and experimental verification of 2FSK modulation

**Figure 4:**
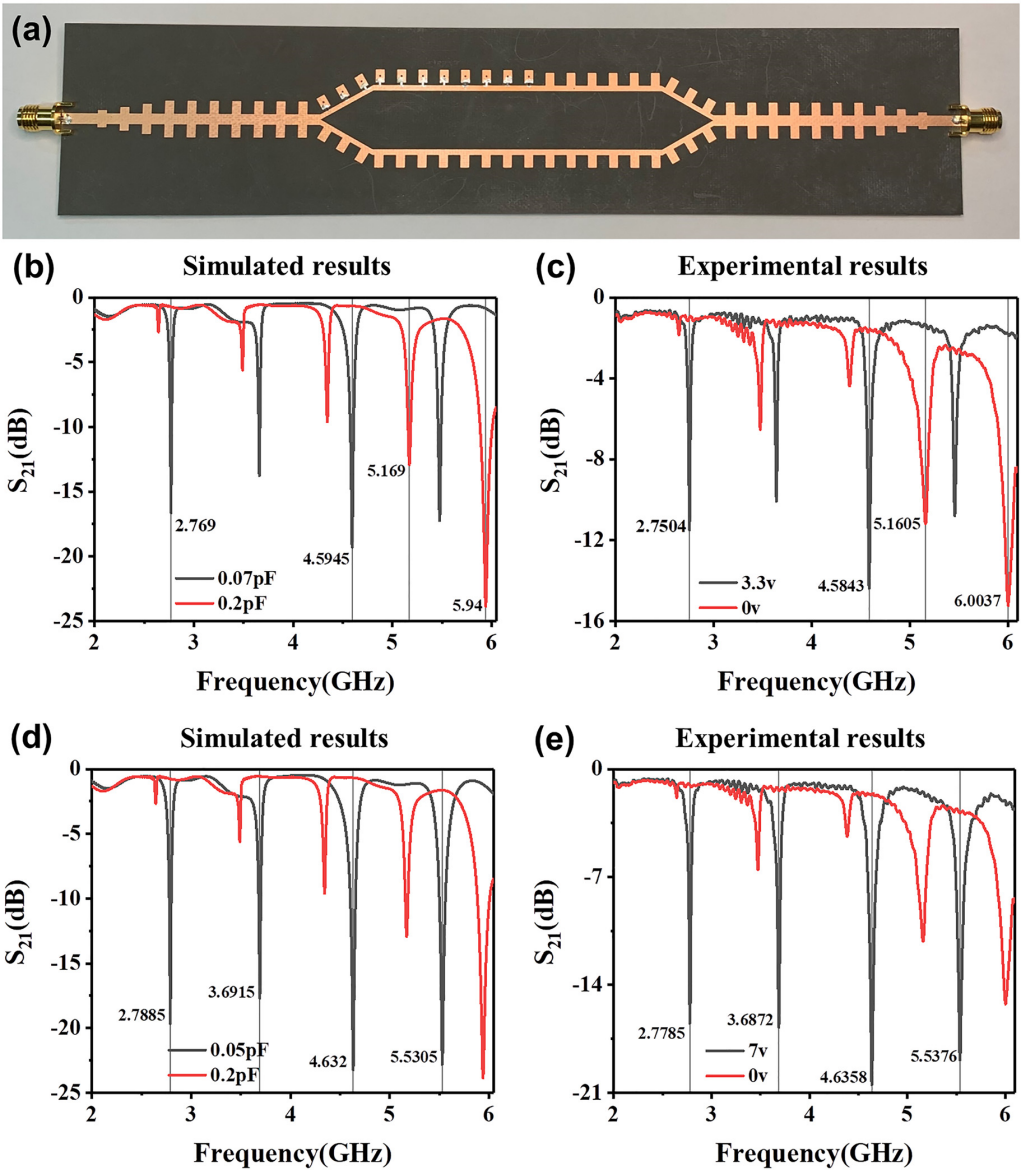
The fabricated reconfigurable SSPP-based MZI and measured results. (a) Photo of the reconfigurable SSPP-based MZI structure shown in Figure 2(b). (b) The simulated S_21_ curves with capacitance 0.07 pF and 0.2 pF and (c) their corresponding experimental results. (d) The simulated S_21_ curves with capacitance 0.05 pF and 0.2 pF and (e) their corresponding experimental results.

According to the above discussions, we fabricated the reconfigurable SSPP MZI structure with 11 diodes, as shown in Figure 2(b), and evaluated its 2FSK performance by simulations and experiments. Figure 4 shows the simulated and measured S_21_ curves under three different bias voltages. The measured spectral positions of the interference dips agree well with the simulation results. The slight difference between the simulated and measured transmission amplitudes may be caused by the fact that the exact capacitance and loss of the varactor diode corresponding to the applied voltage cannot be accurately estimated in the simulations. The relationship between the capacitance and bias voltage is given in the Supplementary Materials.

As shown in Figure 4(b) and (c), when the applied voltage rises from 0 to 3.3 V, we observe a distinct change in the spectrum. At 2.75 GHz, for example, S_21_ is below −11 dB under the bias voltage of 3.3 V and higher than −1.85 dB at 0 V bias. It indicates that the transmission state of the SSPP wave changes from the “on state” to the “off state” when the bias voltage changes from 3.3V to 0 V. Here, we marked four interference dips at 2.75 GHz, 4.58 GHz, 5.16 GHz and 6 GHz, at which the transmission states can be switched by varying the bias voltages. This feature can be leveraged for 2FSK, where the carrier signals overwhelmingly transmitted at one frequency (*f*_1_ = 2.75 or 4.58 GHz) are mostly suppressed at the other frequency (*f*_2_ = 5.16 or 6 GHz), and vice versa. The input binary sequence can be used to manipulate the bias voltage applied on the MZI structure through the digital control system, allowing the frequencies of carrier signals to be switched between *f*_1_ and *f*_2_. It is worth noting that the two carrier frequencies selected for the 2FSK modulation can be very close because of the high *Q* factor of our MZI structure, allowing for high spectrum usage of the communication. Moreover, the frequency difference between two carrier signals can be further reduced by changing the bias voltages. Further increasing the voltage can slightly modulate the center frequency of the interference dips and may improve the depth of the interference dips as well. As shown in Figure 4(d) and (e), when the applied voltage increases to 7 V, all interference dips become deeper, yielding more frequency points that can be regarded as the cutoff state and more selectable frequencies for 2FSK. In addition, the spectral positions of the interference dips can be designed by changing the arm length of MZI, so that 2FSK can be realized at arbitrary frequencies as desired in specific applications. We remark that the reflection property also shows the interference pattern, which is tunable with the biased voltage (detailed discussions are provided in the Supplementary Materials).

**Figure 5:**
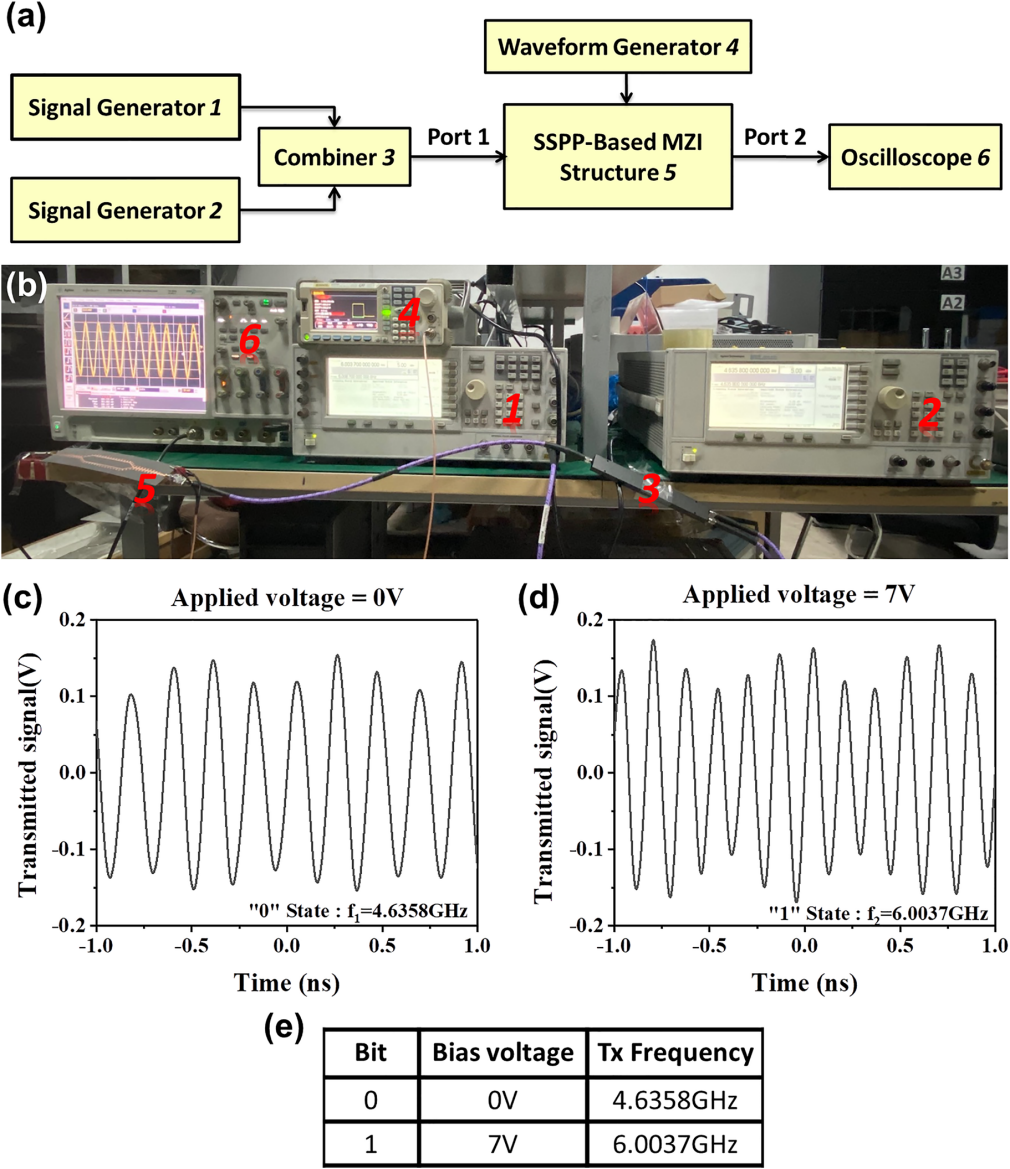
The setup of 2FSK modulation system and measured results. (a) The schematic diagram of measurement system. (b) Photo of the experimental setup. (c)–(d) The measured waveforms under the applied voltages of (c) 0 and (d) 7 V. (e) Table of correspondence among bit, bias voltage and *Tx* frequency.

We also experimentally verified the feasibility of the 2FSK modulations. As shown in Figure 5(a) and (b), two signal generators are used to provide the carrier wave signals with frequencies *f*_1_ and *f*_2_, respectively, and the function/arbitrary waveform generator connected to the SSPP MZI produces pulse waves with different duty cycles to tune the bias voltages. Since the maximum frequency of the signals produced by the waveform generator is farther smaller than that of the carrier waves, we cannot clearly observe the two states of 2FSK signals on one waveform at the same time. Thus, we use a 1 Hz pulse wave to modulate the transmitted signals and record the waveforms in the two states separately. Figure 5(c) and (d) display the measurement results of the example, where the “0” and “1” states of the pulse waves correspond to 0 and 7 V of the bias voltages, respectively, and the two carrier wave frequencies are *f*_1_ = 4.6358 and *f*_2_ = 6.0037 GHz. As the state switches between “0” and “1”, the transmission frequency also switches between 4.6358 GHz and 6.0037 GHz.

## 5 Experimental verification of the amplitude modulation

**Figure 6:**
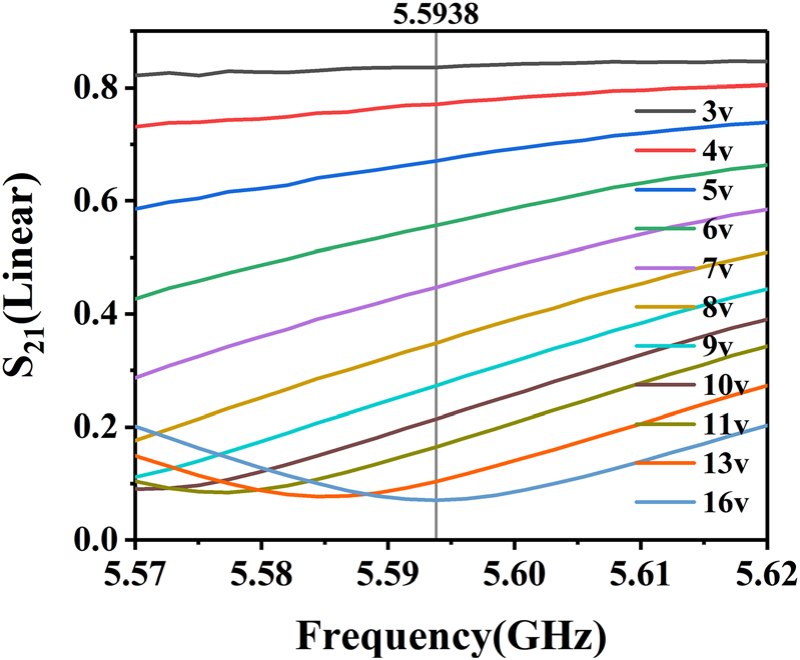
The experimental S_21_ curves for the reconfigurable SSPP MZI structure shown in Figure 2(b) with the bias voltage varying from 3 to 16 V.

The reconfigurable MZI structure can not only be used to achieve 2FSK, but also applicable for the amplitude modulation. At a specific frequency, the amplitude of signal transmission can be continuously tuned via the applied voltage. Figure 6 takes the transmission coefficient change at 5.5938 GHz as an example to demonstrate experimentally the amplitude modulation ability of the SSPP MZI structure. As the applied voltage decreases from 16 to 3 V, the transmission coefficient varies from 0.07 to 0.84, giving an amplitude change of 0.77. In the experiments, the voltage change step can be tuned to only 0.1 V so that the transmission amplitude is varied almost continuously between 0.07 and 0.84. Compared with the 2ASK methods which can only switch between the “on” and “off” states [31, 32], our design enables continuous modulations of the amplitude and thus is applicable for multi-bit encoding in the wireless communications.

**Figure 7:**
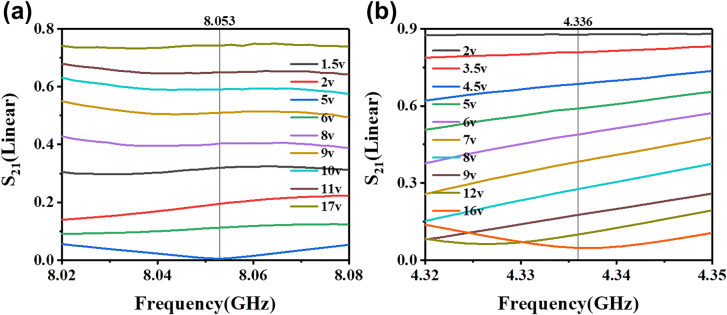
The S_21_ curves at (a) 8.05 GHz and (b) 4.34 GHz of the new SSPP-based MZI structure with *h* = 4 mm and *h*_*2*_ = 3.5 mm at different applied voltages.

The amplitude modulation performance of our MZI structure can be further improved by adjusting the geometric parameters of the SSPP unit. We find that increasing *h* and *h*_*2*_ to 4 and 3.5 mm, respectively, will further improve the modulation depth. In addition, the number of the varactor diodes can be reduced to 9 without compromising the performance. The multiple interference peaks and valleys allow the amplitude modulation at multiple frequencies. As shown in Figure 7(a) and (b), at 8.053 GHz, when the applied voltage is 17 V, the transmission coefficient is 0.74. As the applied voltage is switched to 5 V, the transmission coefficient can be modulated to 0.005, indicating that no energy can be transmitted and the phase difference between the sensing and reference arms is almost 180°. At 4.336 GHz, the transmission coefficient can be continuously modulated from 0.05 to 0.88 by varying the bias voltage from 2 to 16 V within the working range of the varactor diodes, as shown in Figure 7(b).

## 6 Conclusions

We have demonstrated a reconfigurable SSPP-based MZI structure as a new approach to realize the amplitude and frequency modulations. By loading the varactor diodes on the upper arm, the MZI structure becomes asymmetric and the EM waves propagating along the two arms will be interfered. The interference patterns can be dynamically modulated by changing the diode capacitance, and thus tuning the applied bias voltage on the diodes can control the transmission properties. The switchable spectral position of the multiple interference dips allows 2FSK as well as continuous amplitude modulations to be achieved at multiple frequencies on the same device, reaching the reconfigurable capability. The closely located interference dips with high *Q*-factors may be adopted for multi-channel communications with high channel capacities. Our reconfigurable MZI structure with the continuous amplitude modulations has also great potentials for multi-bit encoding, offering a simple and novel solution to multi-scheme modulations for modern wireless communications.

## Supplementary Material

Supplementary Material
