# Supplementary material for: Reconfigurable Mach–Zehnder interferometer for dynamic modulations of spoof surface plasmon polaritons
Source: Nanophotonics. 2021 Dec 1;11(9):1913–21. doi: 10.1515/nanoph-2021-0539 (PMC11501307; doi:10.1515/nanoph-2021-0539)
Supplement: Supplementary file 1 — Supplementary Material [file j_nanoph-2021-0539_suppl.docx]

***Supporting Information for***

Reconfigurable Mach-Zehnder interferometer for dynamic modulations of spoof surface plasmon polaritons

Wen Yi Cui^1,2^, Jingjing Zhang^1,2,3*^, Xinxin Gao^1,2^, and Tie Jun Cui^1,2,3,*^

^1^ *State Key Laboratory of Millimeter Waves, Southeast University, Nanjing 210096, China*

^2^ *Institute of Electromagnetic Space, Southeast University, Nanjing 210096, China.*

^3^ *Center for Intelligent Metamaterials,* *Pazhou Laboratory, Guangzhou 510330, China.*

* Correspondence should be addressed to Jingjing Zhang and Tie Jun Cui. Emails: zhang[jingjing@seu.edu.cn](mailto:jingjingzhang@seu.edu.cn); [tjcui@seu.edu.cn](mailto:tjcui@seu.edu.cn)

1. Relationship between the capacitance of the varactor diode and the biased voltage in experiments

The chart of the capacitance versus bias voltage from the datasheet of the varactor diode is provided in Figure S1. We have also summarized the relationship between the capacitance and bias voltage obtained by fitting the measured and simulated data, as shown in Table S1. Note that the fitted capacitance data in the table are slightly smaller than the given data in Figure S2 but the difference is within the margin of error.


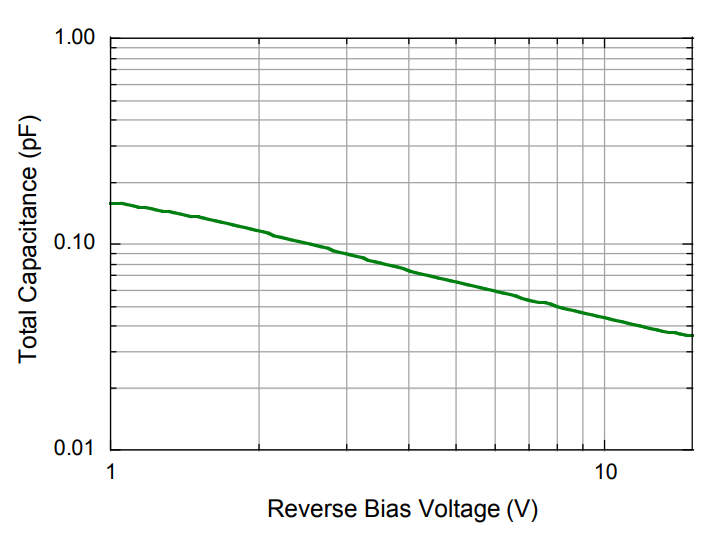


**Figure S1.** Performance curve at 25℃ from the data sheet for the varactor diode MAVR-011020- 1411

**Table S1:** Table of capacitance vs. bias voltage

| **Bias voltage** | **0V** | **3.3V** | **7V** |
| --- | --- | --- | --- |
| **Capacitance** | **0.2pF** | **0.07pF** | **0.05pF** |

1. Reflection property of the SSPP-based MZI structure

The reflection is mainly caused by the interference at the connecting points of two MZI arms. As shown in Figure S2(a), the measured S_11_ curves of the SSPP-based MZI structure also show obvious interference patterns, which are tunable through the variation of the applied voltage. The comparison of S_21_ and S_11_ at the biased voltage of 17V is given in Figure S2 (b), where we can observe that the small transmission at the S_21_ dips is mainly caused by the reflection. The varactor diodes also induce some reflection, but the influence is very small.


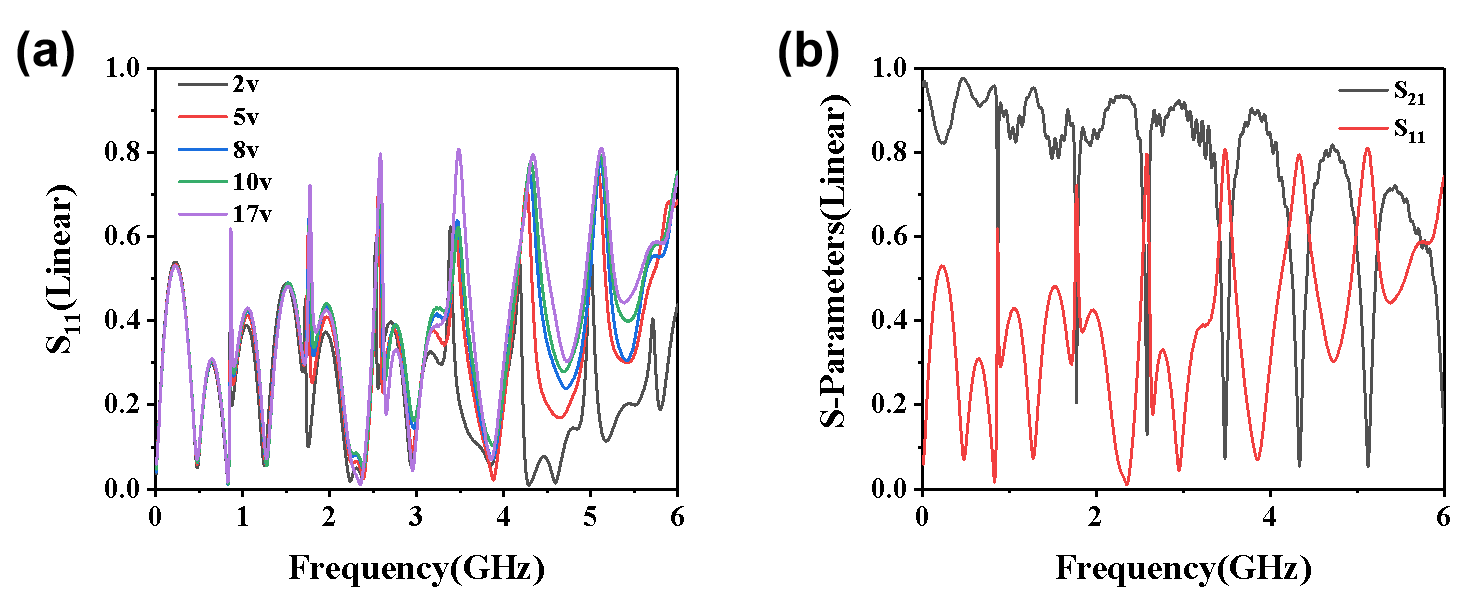


**Figure S2.** (a) S_11_ curves at different applied voltages. (b) S parameters of the SSPP-based MZI structure with *h* = 4mm and *h_2_* = 3.5mm at the biased voltage of 17V.
